# Artificial intelligence for personalized multiple micronutrient supplementation in maternal health

**DOI:** 10.1002/ijgo.70911

**Published:** 2026-02-26

**Authors:** Gabriel Davis Jones, Aris T. Papageorghiou, Hassan Shehata, Hema Divakar, Eline M. Van der Beek, Vyta Senikas, Justin C. Konje, Anne‐Beatrice Kihara, Moshe Hod, the FIGO Committee on Women's Health and Technology

**Affiliations:** ^1^ Oxford Digital Health Labs, Nuffield Department of Women's and Reproductive Health University of Oxford Oxford UK; ^2^ Nuffield Department of Women's and Reproductive Health University of Oxford Oxford UK; ^3^ Oxford Maternal and Perinatal Health Institute (OMPHI) University of Oxford Oxford UK; ^4^ Royal College of Obstetricians and Gynaecologists London UK; ^5^ Divakars Speciality Hospital Bengaluru India; ^6^ Department of Pediatrics, University Medical Centre Groningen University of Groningen Groningen the Netherlands; ^7^ Nestlé Institute of Health Sciences, Nestlé Research Lausanne Switzerland; ^8^ Faculty of Medicine McGill University Montreal Quebec Canada; ^9^ Feto Maternal Centre Doha Qatar; ^10^ Department of Obstetrics and Gynecology, Weill Cornell Medicine Doha Qatar; ^11^ Department of Health Sciences University of Leicester Leicester UK; ^12^ Department of Obstetrics & Gynaecology University of Nairobi Nairobi Kenya; ^13^ Sackler Faculty of Medicine Tel Aviv University Tel‐Aviv Israel

**Keywords:** artificial intelligence, digital maternal health, low‐ and middle‐income countries, maternal nutrition, multiple micronutrient supplementation, nutritional digital twin, personalized nutrition, risk stratification

## Abstract

Maternal undernutrition and micronutrient deficiencies remain pervasive, contributing to adverse pregnancy outcomes and long‐term health risks for mothers and offspring. Multiple micronutrient supplementation (MMS) during pregnancy has demonstrated benefits, including reduced risks of low birth weight, small‐for‐gestational‐age births, and neonatal mortality, when compared with standard iron–folic acid supplementation. Current MMS strategies, however, often follow a standard MMS, overlooking variations in nutritional status, health profiles, and context. Advances in artificial intelligence (AI), particularly deep learning and natural language processing, provide opportunities to strengthen maternal nutrition programs by integrating diverse data sources. Rather than promising fully individualized recommendations, AI could help stratify women by risk of insufficiencies or deficiencies, highlight groups most likely to benefit from additional support, and inform the design of more responsive supplementation strategies during preconception and pregnancy. We outline a conceptual model in which multimodal health data—including electronic health records (EHRs), wearable sensor outputs, nutrition and fertility app logs, genomic markers, and sociodemographic information—are aggregated and analyzed by AI systems to inform personalized MMS plans. The framework introduces the concept of a “nutritional digital twin,” a virtual profile of the patient's nutritional and metabolic state. This digital twin can simulate micronutrient needs and predict maternal–fetal outcomes under different supplementation scenarios, enabling clinicians to test scenario‐based options (e.g. standard MMS ± targeted add‐ons) for individuals. We describe how deep learning models can identify complex patterns (e.g. diet–genome interactions or behavioral trends) while natural language processing (NLP) algorithms extract clinically relevant insights from unstructured data (such as medical notes or patient queries). In addition, we discuss the role of digital maternal health tools, such as mobile apps and wearable trackers, in supplying real‐time data to the AI models and in engaging women to improve adherence to supplementation regimens. Harnessing AI for MMS could transform maternal nutrition care in both high‐ and low‐resource settings. In high‐income contexts, rich data (comprehensive EHRs, genetic tests, continuous monitoring devices) could feed advanced predictive models to support risk‐stratified care with protocolized supplementation options, under clinical oversight. In low‐ and middle‐income countries, where maternal undernutrition and micronutrient gaps are most prevalent, AI‐driven approaches can help stratify risk groups and optimize limited resources. Ubiquitous mobile phone access and digital health tools in many such settings provide avenues for data collection and intervention delivery. We highlight examples where machine learning on population data revealed “hidden hunger” patterns and key predictors of low supplement uptake (e.g. low education, minimal antenatal visits)—insights that policymakers can use to target nutrition programs. A nutritional digital twin could further allow scenario‐testing (e.g. predicting the impact of adding a vitamin D supplement for a specific patient) before clinical decisions are made. To realize this vision, the key concerns are ethics, credibility, and fairness. Ethical frameworks must guide development so that sensitive reproductive health data are protected and clinician oversight remains central. The credibility of AI‐generated recommendations depends on transparency about the assumptions used to translate nutritional and health data into supplement type and dose, and on prospective validation against maternal and neonatal outcomes. This requires a continuous feedback loop in which recommendations are tested in real‐world settings and recalibrated using outcomes data, ensuring that the system learns from observed benefits and harms, rather than relying solely on theoretical modeling. Fairness demands that training data sets represent diverse populations and that solutions are tailored to local contexts to reduce bias and avoid widening disparities. Critically, the approach must be fed by data streams that extend beyond initial demographics and clinical baselines to include biomarkers, adherence patterns, and pregnancy outcomes, so that the models can be refined and dosing rules adjusted over time. If these safeguards are embedded, AI‐enhanced personalized MMS can move beyond proof of concept towards a credible, equitable, and empirically grounded contribution to global maternal health. AI‐driven personalized nutrition support represents a frontier in obstetric care. By combining clinical knowledge with data‐driven intelligence, we can move beyond generalized prenatal supplements towards precision maternal nutrition. The integration of deep learning models and digital health innovations into antenatal care pathways has the potential to better nourish pregnancies, save lives, and ensure healthier futures for mothers and children worldwide.

## 1 INTRODUCTION

Maternal nutrition is a cornerstone of safe motherhood and healthy child development. The “first 1000 days,” spanning from conception to a child's second birthday, represents a critical window in which nutritional status shapes lifelong growth, development, and disease risk.^1^ In many regions, women experience multiple micronutrient deficiencies, often termed “hidden hunger,” due to inadequate dietary intake, infections, or high physiological demands.^2^, ^3^ These deficiencies are associated with anemia, pre‐eclampsia, impaired fetal growth, preterm birth, and other adverse outcomes, such as increased susceptibility to infection and poor maternal recovery.^4^, ^5^ Recognizing these risks, global health experts have investigated antenatal multiple micronutrient supplementation (MMS) as an enhancement over the standard iron and folic acid (IFA) pills given during pregnancy in low‐ and middle‐income countries (LMICs).^6^, ^7^

A robust body of research supports the superiority of MMS over IFA alone in improving pregnancy outcomes.^7^ Meta‐analyses of trials in LMICs have found that MMS significantly reduces the risk of low birth weight by approximately 14% and of small‐for‐gestational‐age newborns by approximately 8%.^6^ A 2017 individual patient‐data meta‐analysis of 17 trials (>112 000 women) reported that MMS led to lower odds of low birthweight (relative risk [RR] ~0.81) and fewer SGA births compared to IFA.^7^ MMS benefits were particularly pronounced in high‐risk groups: women who were undernourished (low body mass index [BMI, calculated as weight in kilograms divided by the square of height in meters]) or anemic saw greater improvements in birth outcomes and infant survival. Starting supplements early (preferably in the first trimester, but if not, before 20 weeks) and achieving high adherence conferred additional risk reductions in preterm birth.^8^ Notably, these gains were achieved without any increase in adverse outcomes; studies show MMS does not raise the risk of stillbirth or neonatal mortality overall. On the contrary, some evidence suggests MMS may reduce neonatal mortality, particularly among female infants, and improve infant survival up to 6 months in populations of women with poor nutritional status.^7^ These findings have spurred calls to update antenatal care guidelines. Whereas past WHO recommendations were cautious about universal MMS (partly due to cost considerations), new analyses argue that replacing IFA with MMS could prevent hundreds of thousands of babies from being born with low birth weight each year.^9^ Several countries have begun exploring adopting MMS in routine prenatal programs.^10^, ^11^

However, even with the adoption of universal MMS policies, not all women may have the same micronutrient needs.^12^ Nutritional requirements can differ based on a woman's baseline status (e.g. an adolescent mother vs. an older multigravida), co‐existing morbidities (such as tuberculosis, malaria, or HIV, which affect nutrient metabolism), genetic factors (e.g. influencing folate or vitamin D processing), BMI, nutritional status, and dietary intakes.^13^, ^14^ Adherence also varies: some women may not take supplements regularly due to anticipated side effects, forgetfulness, or low awareness of benefits.^15^ Simply providing every pregnant woman the same MMS may therefore be adequate for some, but either unnecessary for others or insufficient for those with higher requirements.

Integrating artificial intelligence (AI) into maternal nutrition programs offers a pathway to address heterogeneity by leveraging diverse data streams such as demographic characteristics, anthropometric data, clinical history, dietary assessments, and biomarker profiles to generate risk profiles and guide supplement strategies.^16^ In practice, such approaches will initially be most feasible for pregnant women already engaged with antenatal care systems, since these women provide the structured data needed to inform models. Machine learning algorithms could identify which women are most likely to benefit from additional nutrients or targeted interventions, and digital health platforms may help to detect barriers to adherence in real time. By combining predictive modeling with decision‐support tools embedded in routine antenatal care, AI systems could assist healthcare providers in tailoring supplement composition, dosing schedules, and counseling strategies to the specific stage of pregnancy, distinguishing between early and later needs. Ultimately, this risk‐stratified approach aims to improve maternal and fetal outcomes by ensuring women receive appropriate nutrients at the most relevant time.^17^, ^18^, ^19^

In this paper, we explore four prospective applications of AI in MMS. We first detail methods by which machine learning and natural language–processing techniques can integrate electronic health records (EHRs), wearable and app‐derived data, and, where available, genomic and metabolomic profiles to estimate individual micronutrient requirements. We then introduce the concept of a nutritional digital twin: an in silico model that simulates maternal–fetal nutrient dynamics under different supplementation regimens and can guide regimen selection. We next examine population‐level approaches, including unsupervised clustering to identify subgroups at elevated risk of deficiency and predictive modeling to inform resource allocation and policy decisions. Finally, we consider practical deployment via digital health data platforms and address the accompanying ethical, equity, and scalability challenges inherent in implementing AI‐driven personalized MMS across diverse health systems.

## 2 AI‐DRIVEN FRAMEWORK FOR PERSONALIZED MMS

**FIGURE 1:**
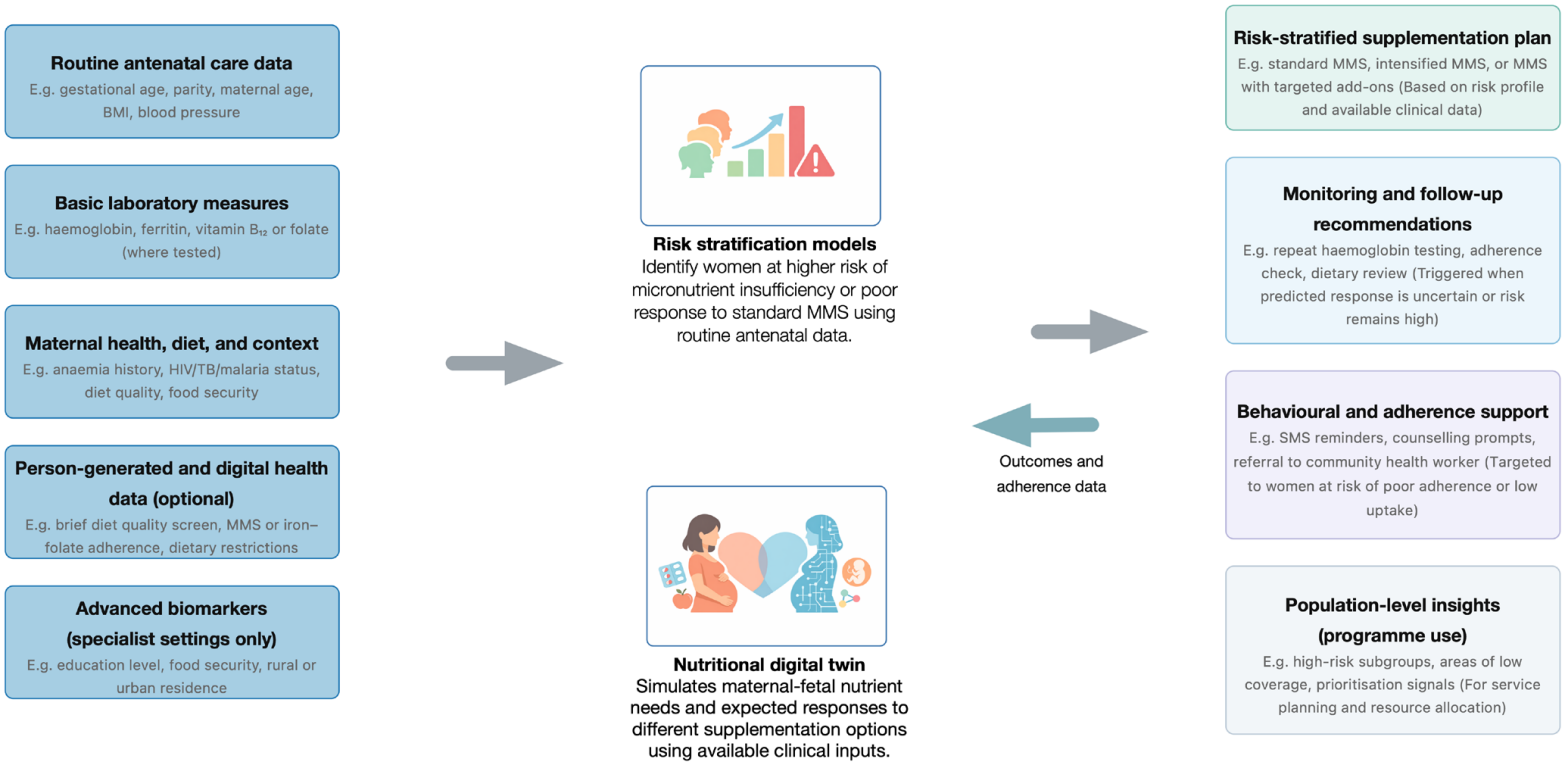
Conceptual framework for AI‐guided, risk‐stratified multiple micronutrient supplementation in pregnancy. Routinely collected antenatal care data and basic laboratory measures form the core inputs to the system, with additional contextual, person‐generated, digital, and specialist biomarker data incorporated where available. Risk stratification models identify women at higher risk of micronutrient insufficiency or suboptimal response to standard MMS, while a nutritional digital twin simulates maternal–fetal nutrient needs and expected responses under different supplementation scenarios using available clinical inputs. Model outputs support clinician‐reviewed decisions on risk‐stratified supplementation plans, monitoring and follow‐up, targeted behavioral and adherence support, and population‐level insights for program planning. Longitudinal pregnancy outcomes and adherence data feed back into the models to enable ongoing refinement, while defaulting to standard MMS when uncertainty is high. MMS, multiple micronutrient supplementation.

In our proposed framework, EHRs provide the foundation for improved micronutrient recommendations by combining structured laboratory values with information extracted from clinician notes (Figure 1). Techniques in natural‐language processing enable the identification of dietary restrictions or surgical histories relevant to nutrient absorption, such as bariatric procedures or vegan diets, which would otherwise remain hidden in unstructured text.^20^, ^21^, ^22^ These enriched records allow predictive models to flag individuals at risk of specific deficiencies, for example recognizing a pattern of low serum vitamin B_12_ alongside documented dietary avoidance and suggesting an adjusted supplementation regimen.

Wearable devices and ambient sensors offer complementary insights into a woman's physiological status throughout pregnancy. Continuous monitoring of activity levels, heart rate variability, and sleep patterns can reveal early signs of fatigue or circulatory changes that correlate with anemia or thyroid dysfunction.^23^, ^24^, ^25^ When integrated with clinical data, these real‐time measurements permit dynamic risk stratification, so that emerging nutritional concerns (such as a sudden decline in daily step count paired with weight stagnation) can prompt targeted dietetic support or modification of supplement doses.

Self‐reported data from nutrition and fertility applications can add useful contextual information by capturing daily intake, symptoms, and adherence behaviors; however, most consumer‐oriented apps lack validation and are not scientifically robust. Tools designed for research settings can provide greater accuracy, yet they usually demand considerable effort from participants, and all approaches depend on the availability of reliable food composition databases as well as accurate reporting of portion sizes. These limitations highlight the need for continued improvement in data capture methods, integration of standardized nutrient databases, and validation of consumer‐friendly platforms before they can be relied upon in clinical decision‐making. Nonetheless, aggregating food logs through AI algorithms offers the potential to identify dietary gaps, e.g. persistently low intake of calcium‐rich foods despite use of prenatal vitamins, and to highlight where supplementation or dietary counseling may be warranted. Such platforms can also act as intervention channels, delivering tailored reminders or educational messages that reinforce adherence and enable monitoring of responses over time.^26^, ^27^

Genetic and metabolomic profiles introduce a deeper layer of individual variation that can refine supplementation strategies in high‐risk or specialized settings. Polymorphisms in genes such as MTHFR have well‐documented effects on folate metabolism, and metabolite panels can reveal subclinical deficiencies not evident in routine blood tests.^14^ By incorporating these high‐dimensional data into machine learning models, the framework can suggest alternative formulations, such as methyl folate rather than folic acid, or additional micronutrients based on a patient's unique biochemical signature.

Sociodemographic and environmental variables complete the data mosaic by contextualizing individual needs within broader determinants of health. Factors including age, parity, level of education, household food security, and local prevalence of infections influence both baseline nutrition and supplement uptake.^5^ Machine learning analyses of large survey data sets have demonstrated that women in certain socioeconomic strata or geographic regions are disproportionately unlikely to adhere to supplementation, insights that can guide the deployment of community health workers or resource allocation.^28^

Data quality will be crucial as a key driver for the reliability of any AI‐driven recommendations. Self‐reported dietary data through mobile applications may have limitations, such as portion size estimation errors, social desirability bias, and incomplete food database coverage. Consumer wearables that provide continuous data streams for parameters like energy expenditure and sleep quality may perform differently across populations. EHRs, though often more reliable, may contain incomplete nutritional assessments or only point‐in‐time snapshots that fail to capture dynamic changes. Even genomic data require careful interpretation, as gene–nutrient interactions can vary by background and environmental context. To mitigate these challenges, a pragmatic minimum data bundle for safe use should be considered. This could include gestational age, hemoglobin with ferritin where available, BMI, HIV/tuberculosis/malaria status, and a brief dietary screen or adherence proxy, with additional markers such as 25‐hydroxy vitamin D and B_12_/folate testing, where feasible. When these inputs are not available, the system should default to recommending standard MMS rather than attempting risk stratification. Such safeguards would reduce the risk of systematic biases, while uncertainty quantification and clear communication of confidence levels would remain essential for any AI‐driven guidance.

For operational safety and equity, we propose a pragmatic, tiered data policy. At the basic level, the system requires a minimum data bundle that clinics routinely collect: gestational age, hemoglobin, BMI, and a question regarding adherence. Where those data are the only inputs, the system defaults to recommending standard MMS and uses clinic‐held pseudonymized identifiers to permit pseudonymized longitudinal tracking of hemoglobin and pregnancy outcomes. At intermediate levels, the addition of ferritin or simple point‐of‐care tests refines risk stratification. Only in settings with reliable laboratory or genomic access would the system use advanced biomarker inputs to change formulations. This tiered design ensures the model never withholds standard care because diagnostics are unavailable, while allowing follow‐up and audit without exposing patient identities.

To synthesize these heterogeneous inputs, multimodal deep learning architectures learn latent representations that capture interactions between clinical measurements, sensor streams, self‐reports, and external characteristics. The resulting models output quantitative risk scores and personalized supplement compositions with accompanying explanatory highlights, e.g. indicating supplementation options that move standard MMS to MMS with iron intensification due to a combination of low hemoglobin, decreased activity, and limited dietary intake. Crucially, these outputs are intended to support rather than supplant clinical judgment, presenting results in accessible formats for both practitioners and policymakers, and enabling proactive identification of at‐risk populations before adverse outcomes occur.

**Case scenario**

Amina is a rural, first‐trimester woman who attends a clinic equipped with a digital antenatal registry and uses a basic smartphone app. At her initial visit, blood tests reveal moderate anemia and she reports frequent dizziness. Dietary recall logged in the app shows low protein intake, and her farm work implies high energy expenditure. An AI system integrates these data with her clinical history and classifies her as high risk for iron and vitamin B_12_ insufficiency. The system recommends a standard MMS formulation supported with additional dietary advice to increase intake of iron‐ and protein‐rich foods, and it initiates an SMS‐based reminder program. Because anemia is not always corrected by iron alone, the system flags the need for close follow‐up to assess whether changes in hemoglobin reflect improved intake or whether other factors are contributing, such as gastrointestinal absorption problems. The app continues to monitor her dietary logs so that declining intake of relevant food groups can be identified and addressed. As she uses a low‐cost wearable wristband, declining activity levels or persistent symptoms trigger alerts that prompt a community health worker to follow up. Over time, the AI model re‐evaluates her status and may suggest adjunct measures, such as fortified snacks or referral for further investigations, if adherence is high but clinical indicators fail to improve. This continuous feedback loop illustrates a shift from uniform supplementation to adaptive, data‐informed nutrition management that combines supplementation, dietary guidance, and human oversight. In settings such as Amina's, the system would run in its basic tier: clinic staff use a pseudonymized registry and SMS‐based reminders so that outcomes and hemoglobin trends can be followed over time without storing identifying data centrally.

## 3 ‘NUTRITIONAL DIGITAL TWIN’

### 3.1 A virtual mirror for maternal nutrition

Central to our proposal is the nutritional digital twin, a computational model that mirrors the physiological processes of mother and fetus to predict responses to micronutrient interventions. Digital twins have been adopted in cardiology and metabolic disease to simulate organ function and personalized nutrition, demonstrating their ability to forecast individual outcomes and guide treatment choices.^29^, ^30^, ^31^ In the maternal context, the twin would assimilate a woman's clinical measurements, anthropometric data—such as pre‐pregnancy BMI—and patterns of gestational weight gain, and, where available, measures of body composition that provide more refined insights into nutritional status. These inputs, combined with dietary records, wearable sensor data, and genomic or metabolomic profiles, would form an integrated physiological framework capable of modeling maternal–fetal nutrient dynamics and forecasting outcomes under different supplementation strategies.^29^, ^30^, ^31^

The digital twin is designed in two practical modes. The full twin uses repeated biochemical and sensor data to simulate maternal–fetal nutrient dynamics. A simplified twin uses only routinely available clinical inputs (gestational age, hemoglobin, BMI, and a short diet screen) to generate conservative risk estimates and scenario comparisons. The simplified twin is intentionally conservative: when uncertainty is high it recommends standard MMS and closer monitoring rather than experimental dosing. Validation should proceed in stages, first comparing simplified twin outputs with observed hemoglobin and birthweight in prospective cohorts, then validating the full twin in nested studies where more data are available.

This virtual representation comprises interconnected modules for maternal nutrient stores, placental transfer dynamics, and fetal growth trajectories. As new information becomes available, such as hemoglobin measurements or activity trends, the model updates its internal parameters and refines predictions. Clinicians could then pose “what‐if” scenarios, for example evaluating the likely change in fetal growth when moving from standard MMS to MMS and iron intensification, or simulating the impact of deworming on maternal iron status by the third trimester.^32^

A nutritional digital twin offers applications before, during, and after pregnancy, across the continuum of care. In preconception counseling, it can identify nutrient deficits to correct before conception, although this is only feasible if women engage with health services early. In many contexts, women present late for their first antenatal visit, limiting opportunities to influence outcomes that depend on early placental development. When data are available, early in gestation, the twin may help stratify women by risk of anemia or impaired placental and fetal growth, thereby informing the intensity of supplementation. Mid‐pregnancy adjustments could be guided by simulated outcomes under alternative dosing regimens, while late‐gestation predictions might trigger additional monitoring or interventions when the twin forecasts elevated risk of preterm birth. Postpartum, it could support recovery, including weight loss and replenishment of nutrient stores, which may be especially relevant in cases of complications, such as gestational diabetes and during breastfeeding. Aggregated across cohorts, these models could also provide policymakers with simulated population‐level outcomes under different supplementation strategies.

Realizing this vision requires high‐quality longitudinal data, rigorous validation, and careful translation into user‐friendly interfaces. Data scarcity and heterogeneity, especially in low‐resource settings, remain major challenges for training robust models, and clinical uptake will depend on showing that twin‐based recommendations improve outcomes without adding unnecessary complexity. Early digital‐twin initiatives in diabetes and cardiology underscore the feasibility of blending data‐driven learning with established nutritional science.^33^, ^34^ However, building a solid approach across the continuum of care will also require concerted efforts to engage women early in pregnancy, or ideally before conception, so that critical windows for placental and fetal development are not missed. Within these constraints, a maternal nutritional digital twin offers a potentially scalable tool to support micronutrient optimization and bring greater precision to prenatal care.^33^, ^34^

**Case scenario**

Consider Sara, a 28‐year‐old woman attending her first antenatal visit, which, in many LMICs, may occur anywhere between 10 and 20 weeks of pregnancy. At this visit, her baseline data include a hemoglobin of 100 g/L, a diet diary showing low iron and vitamin B_12_ intake, and genetic screening identifying an MTHFR variant. These data are fed into her nutritional digital twin. Over the next 4 weeks, the twin integrates new hemoglobin measurements and activity data from her wristband, recalibrating its internal model. When the twin simulates stepping up to iron intensification, it forecasts a rise in hemoglobin to 115 g/L by 28 weeks and predicts normal fetal growth percentiles, while standard dosing would leave her at risk of persistent anemia. Guided by these insights, her clinician prescribes and supervises the adjusted regimen, with follow‐up twin updates confirming the expected improvements. This example illustrates how an in silico pregnancy model can adapt to the timing of care entry and still inform supplementation strategies to optimize outcomes.

## 4 THE POWER OF CONNECTED CARE

Health platforms can embed prenatal nutrition support within women's everyday activities by combining continuous data capture, tailored feedback, and targeted outreach. Mobile applications enable photograph‐based meal logging, barcode scanning, and manual entry of supplement intake, from which algorithms estimate dietary patterns and highlight potential gaps, although these tools require validation studies to confirm their accuracy and utility.^35^ Wearable sensors monitor indicators such as physical activity, heart rate variability, and sleep quality; when considered alongside clinical data, these signals may provide early clues to changes in maternal well‐being and nutritional risk.^23^ Importantly, micronutrient status is determined not only by intake but also by maternal nutrient stores, so what matters is the ability to track trends over time and to relate these changes to reported diet and supplementation behavior. Person‐generated health data can be linked to AI systems through secure interfaces, allowing models to reflect evolving maternal status rather than relying only on sporadic clinic measurements. In addition, these platforms can capture the minimum data bundle via checklist prompts (e.g. the FIGO Nutrition Checklist) as a basis for risk stratification.

High‐frequency engagement with mobile reminders and app‐based prompts has been shown to improve adherence to prenatal micronutrient regimens. A systematic review in Global Health Action reported that text‐message interventions increased medication adherence by a median of 14% across maternal health studies, including those targeting supplement use.^36^ In addition, a systematic review by Gomes and colleagues identified SMS reminders as one of several effective strategies, alongside education sessions, free supplement provision, consumption monitoring by community volunteers or family members, and multicomponent community mobilization, with adherence gains in the range of 15%–40% in diverse low‐resource settings.^37^ Together, these findings indicate that digital prompts, when embedded within broader support frameworks, can help overcome common barriers to supplement use such as forgetfulness, side effects, and low health literacy.

Beyond simple reminders, modern applications utilize behavioral science principles.^38^ Personalized push notifications adapt to an individual's routine, for example suggesting a morning dosing time if evening pills are consistently missed. Gamification features reward streaks of supplement adherence, and in‐app peer forums foster community support by enabling women to share experiences and coping strategies.^39^ Clinicians and community health workers access dashboard interfaces that summarize adherence data and trigger follow‐up when users fall below defined thresholds. Such hybrid care models, sometimes termed e‐perinatal clinics, blend automated monitoring with human outreach and have proven feasible in extending support to remote populations with limited access to in‐person care.

Equitable deployment of smart devices requires careful attention to the digital divide. In areas with low smartphone penetration or unreliable Internet, feature‐phone solutions using SMS and interactive voice response deliver personalized prompts and educational messages at scale. Co‐design with end users ensures that interfaces remain intuitive, culturally appropriate, and available in relevant languages. Safeguarding user privacy through transparent consent processes, data minimization strategies, and compliance with frameworks such as the EU General Data Protection Regulation (GDPR) or US Health Insurance Portability and Accountability Act (HIPAA) is essential to maintain trust and protect sensitive reproductive health information.^40^ When these considerations are met, digital maternal health tools serve as both the eyes and ears of AI systems, capturing rich, real‐world data, and as their voice, conveying actionable guidance directly to women and their care teams.

### 4.1 Integrating manual tools with AI: The FIGO Nutrition Checklist

The FIGO Nutrition Checklist, first introduced in 2015, is a brief nutritional questionnaire focusing on BMI, diet quality, and micronutrient intake, designed to rapidly identify dietary risks before and during pregnancy. Studies have shown it is acceptable to both women and providers, easy to complete in less than 2 min, and effective in identifying women with suboptimal dietary quality.^41^, ^42^ It has also been used to initiate conversations about diet and weight management in routine antenatal care and integrated into interventions to improve maternal nutrition and offspring health.^43^, ^44^, ^45^

Although traditionally administered in community or clinic settings by health workers, AI‐enabled digital platforms could expand the reach and functionality of the FIGO Nutrition Checklist. Smartphone applications could adapt the checklist into interactive formats, provide tailored counseling messages, and link responses to digital twins that simulate nutritional needs in ways that are responsive to pregnancy stage and maternal characteristics. Community health workers using tablets could capture checklist data in low‐resource settings, with AI models stratifying women by risk and suggesting context‐specific actions. The effectiveness of such simulations will depend on the quality and type of input data available, such as gestational age, anthropometry, dietary intake, and biomarker results. In this way, AI does not replace proven manual tools but enhances their usability, scalability, and integration into broader antenatal care.

## 5 GLOBAL APPLICABILITY

### 5.1 Bridging contexts and reducing disparities

High‐income countries possess comprehensive prenatal data, from thyroid panels to genomic screens, and many pregnant women use wearable devices. AI models can integrate EHRs, sensor outputs, and genetic profiles to identify those who may require tailored supplementation, such as additional choline for optimal neurodevelopment.^46^ Geospatial analyses of these data sets can further reveal underserved subpopulations—rural communities or ethnic minorities—with poorer nutritional outcomes and guide targeted fortification or free‐supplement programs, although randomized trials are needed to validate benefits over standard care.

In LMICs, undernutrition remains widespread even as mobile connectivity improves. Machine learning work in Bangladesh using Demographic and Health Survey data has achieved over 76% accuracy in predicting pregnant women's nutritional status from demographic, wealth, and regional indicators.^47^ By feeding coverage and adherence metrics from national supplementation campaigns into AI systems, health ministries could dynamically adjust distribution strategies.

Equitable implementation demands AI tools that function offline or on basic smartphones and that leverage community health workers to collect data and convey personalized advice. Interfaces must be localized linguistically and culturally and embedded within public health systems rather than confined to private clinics. Training frontline staff in AI interpretation and investing in digital literacy are essential. When aligned with local infrastructure and needs, AI‐tailored MMS can help narrow global disparities in maternal and neonatal health.

Adoption of MMS itself remains the subject of ongoing policy debate in many countries, shaped by cost, supply chains, and prioritization within essential antenatal care packages. AI‐guided risk stratification should be viewed as complementary rather than competing with universal MMS policies: where MMS is already adopted, AI may help target follow‐up and support; where adoption is partial, it may help policymakers prioritize high‐risk groups within constrained budgets. Positioning AI as a tool for optimization rather than expansion may improve alignment with national maternal health agendas.

## 6 LIMITATIONS

The evidence base for AI applications in maternal nutrition remains limited, with most supporting data drawn from other clinical domains or basic demographic modeling. In addition, technical capacity and infrastructure available in many healthcare settings, particularly those serving the populations with the highest burden of maternal malnutrition, may not yet be ready. Added to this are data availability and quality constraints; continuous wearable monitoring, comprehensive genomic profiles, and detailed dietary tracking are not routinely available in clinical practice, and integration of such data across different health systems, mobile platforms, and laboratory information systems presents substantial technical challenges.

Data scarcity and the uneven availability of diagnostics are core limitations. Our framework explicitly assumes a tiered implementation so that lack of genomic or wearable data does not block safe care: the model falls back to standard MMS when only the minimum bundle is present and flags the need for follow‐up rather than changing doses. Future work must measure whether tiered deployment translates into sustained improvements in care rather than remaining only a data collection exercise.

Nevertheless, our aim is to describe possible future directions. At the same time, we should not divert resources from fundamental interventions, such as ensuring food security, clean water, and basic antenatal care, which remain priorities in many global settings.

Finally, the regulatory and ethical frameworks for AI in health care are still evolving, creating uncertainty about approval pathways and standards for clinical integration. These limitations underscore the need for cautious, evidence‐based development rather than premature widespread deployment.

## 7 ETHICAL, EQUITY, AND SCALABILITY CONSIDERATIONS

Ethical deployment of AI in maternal nutrition demands vigilance against algorithmic bias and strong protection of personal data. Models must be trained on diverse data sets that represent different ethnicities, socioeconomic groups, and care settings, and they should undergo subgroup performance testing to detect and correct unfair predictions.^48^, ^49^, ^50^, ^51^ Transparency in model logic and provision for clinician oversight ensure that recommendations are interpretable and can be overridden when necessary.

Informed consent procedures must specify what personal and health data are collected, how they are used, and with whom they are shared, with strict adherence to data‐protection regulations. Just as importantly, validation requires rigorous testing of AI‐driven recommendations against clinical outcomes in prospective studies, external validation in different populations, and continuous post‐deployment monitoring to confirm that predictions remain accurate, safe, and relevant over time.

To protect anonymity while allowing learning from outcomes, we recommend practical safeguards: local storage of identifying data at the clinic, use of pseudonymized unique identifiers for longitudinal linkage, and de‐identified central analysis that receives only de‐identified summaries. Where connectivity permits, federated learning can be used; models are trained locally on clinic data and only the model updates (not patient records) are shared to improve the central algorithm. For sites without Internet, periodic, manual, encrypted uploads of deidentified outcome summaries can permit audit and model recalibration. These steps balance the need for longitudinal evaluation with strong protections for patient privacy. In all deployment settings, ownership of maternal health data remains with the woman and the healthcare system providing care, not with AI developers or platform vendors. AI tools described here function strictly as clinical decision‐support systems: they generate risk estimates and scenario analyses but do not issue treatment orders. Responsibility for interpretation and final clinical decisions rests with the supervising clinician and healthcare institution, consistent with existing medico‐legal frameworks for decision‐support software. As regulatory pathways mature, AI‐guided MMS tools should be assessed and approved within national digital health and software‐as‐a‐medical‐device frameworks, aligned with guidance from bodies such as WHO, national medicines regulators, and digital health authorities.^52^

Equity of access requires tailoring solutions to local infrastructure and digital literacy. In low‐resource areas, AI tools should function offline or on basic smartphones, with community health workers facilitating data collection and personalized counseling. Interfaces must be available in local languages and designed with input from end users to ensure cultural relevance and usability.^53^ Embedding AI‐driven MMS into public health programs rather than limiting them to private clinics will help to reach the most vulnerable populations and avoid widening existing disparities.

Scalability and sustainability depend on demonstrating cost‐effectiveness and building capacity at all levels. Economic evaluations should compare AI‐guided supplementation against standard care, accounting for downstream savings from improved outcomes. Training for frontline staff in basic AI interpretation and digital skills is essential, as is investment in reliable power and connectivity solutions. Ongoing monitoring and iterative refinement of models—based on real‐world feedback and outcome data—will maintain accuracy and trust, while alignment with national eHealth strategies and regulatory frameworks will support safe, widespread adoption.

Safety monitoring and adverse event detection are critical to mitigate harm from incorrect AI‐based risk stratification. Misclassification could result in under‐treatment if high‐risk women are incorrectly categorized as low risk, or unnecessary exposure if low‐risk women receive intensified supplementation. To address this, the framework relies on conservative defaults, longitudinal hemoglobin and outcome monitoring, and automatic reversion to standard MMS when observed clinical trajectories diverge from predicted responses. MMS must consider relevant conditions such as hemochromatosis, and potential interactions with existing medications or conditions. AI recommendations must incorporate safety thresholds and contraindication screening, with built‐in alerts for scenarios where supplementation could cause harm. Real‐world deployment will therefore also require prospective safety monitoring protocols; such automated detection of adverse events through EHR and patient‐reported outcome measures will act as “safety rails” that default to conservative, clinically established dosing when uncertainty exists.

## 8 CONCLUSION

The integration of AI into multiple micronutrient supplementation programs represents a potentially transformative advance in maternal nutrition. By harnessing clinical data, behavioral insights, and physiological modeling, AI can help move care beyond undifferentiated interventions towards more targeted recommendations. A nutritional digital twin could synthesize EHRs, wearable sensor streams, diet logs, and, where available, genomic or metabolomic profiles into a unified in silico model capable of simulating maternal–fetal responses to varied supplement regimens. Such a tool would not only support adjustments during pregnancy but could also extend into pre‐pregnancy, when optimizing nutritional status is known to improve maternal and neonatal outcomes, and into the postpartum period, particularly for women who are breastfeeding and recovering from pregnancy. Framing supplementation across this broader continuum of care highlights the potential of AI‐driven approaches to strengthen maternal health before, during, and after pregnancy.

Digital health platforms are poised to provide the infrastructure for continuous data capture and patient engagement. High‐frequency prompts, context‐sensitive educational messaging, and behavioral adherence features could increase supplement uptake. Peer support forums and clinician dashboards might facilitate a hybrid model of care in which automated monitoring triggers targeted outreach by community health workers, thus extending antenatal support into underserved populations. In turn, these platforms would generate rich person‐generated health data to refine AI algorithms and personalize interventions further.

AI‐driven personalized MMS could find applicability across diverse resource settings. In high‐income countries, interoperable health records, continuous monitoring devices, and precision‐medicine initiatives could enable targeted supplement formulations, such as higher choline or vitamin D doses for subgroups at risk of neurodevelopmental or skeletal complications. In low‐ and middle‐income contexts, expanding mobile connectivity could allow demographic and survey data to feed high accuracy risk‐stratification. Ensuring equitable implementation will require tools that operate offline or on basic devices, interfaces localized in language and culture, and integration into public health programs with appropriate training and support. By addressing algorithmic bias, safeguarding privacy and investing in capacity building, AI‐enhanced MMS could become a scalable, equitable component of comprehensive maternal care, ultimately improving health outcomes for mothers and their children worldwide.

Although the clinical evidence for MMS over standard IFA supplementation is robust, the evidence base for AI‐guided approaches in maternal nutrition is still nascent. Current applications of AI in nutrition are largely confined to dietary pattern recognition, demographic risk stratification, and adherence monitoring. The more advanced applications proposed in this framework will require careful validation before clinical deployment. Real‐world evaluation should follow concrete steps, including prospective cohort studies with decision‐impact analyses, hybrid effectiveness–implementation trials, and pragmatic cluster randomized controlled trials (RCTs) comparing risk‐stratified MMS against standard care. Importantly, primary outcomes must extend beyond efficacy to include safety monitoring for potential risks such as hypervitaminosis or iron overload, while also assessing adherence, feasibility, and cost‐effectiveness. Engagement of clinicians, data scientists, policymakers, and community representatives will be essential to refine algorithms, ensure ethical deployment, and embed these tools within existing maternal health programs. With sustained investment in research, infrastructure, and workforce capacity, AI‐supported supplementation could ultimately strengthen antenatal nutrition and contribute to reducing maternal and neonatal morbidity and mortality worldwide.

**Research agenda for AI‐driven MMS**

Translating this framework into clinical practice requires a comprehensive research agenda addressing both technical development and clinical validation. Priority areas include:

- Minimum data requirements: What constitutes the minimum safe and effective data bundle (gestational age, hemoglobin ± ferritin, BMI, HIV/tuberculosis/malaria status, dietary screen/adherence proxy, and where feasible 25‐OH vitamin D and B_12_/folate)? How does model performance degrade when only partial data are available?
- Generalizability and equity: Do models perform consistently across diverse populations, including different ethnicities, socioeconomic groups, infection burdens, and healthcare systems? What approaches best detect and mitigate algorithmic bias?
- Study design and validation: Which study designs are most efficient and reliable for evaluation – prospective cohorts with decision‐impact analyses, hybrid effectiveness‐implementation trials, or pragmatic cluster RCTs? How should maternal and neonatal outcomes be prioritized?
- Safety monitoring: How can signals of hypervitaminosis, iron overload, or other unintended harms be detected early and incorporated into model refinement? What safety thresholds should trigger automatic reversion to standard MMS?
- Integration of outcomes data: How should maternal, fetal, and infant outcomes be linked back into model training to create a continuous learning health system?
- Usability and clinical adoption: What interface designs allow frontline health workers and clinicians to understand and act on AI outputs? How do these tools affect workflow, counseling, and trust in recommendations?
- Implementation and scalability: What models of deployment (e.g. integration into public antenatal care, use by community health workers, mobile‐first tools) are feasible in low‐resource versus high‐resource settings?
- Economic evaluation: What are the cost‐effectiveness implications of AI‐driven MMS compared with standard supplementation, accounting for long‐term maternal and child health outcomes?
- Ethical and governance frameworks: How should consent, privacy, and data ownership be managed, particularly in contexts where digital literacy or regulation is limited?
- Evaluate long‐term implementation outcomes: does tiered AI support produce sustained improvements in supplement coverage, hemoglobin trajectories, and birth outcomes compared with standard care in pragmatic trials and routine programmatic rollouts?

Answering these questions will be essential to move beyond proof of concept and determine whether AI‐guided MMS can deliver safe, equitable, and cost‐effective improvements in maternal and neonatal health.

## AUTHOR CONTRIBUTIONS

GDJ conceived the study with MH, conducted the research, and drafted the manuscript. MH contributed to the conception of the study. ATP, HS, HD, EMVdB, VS, JCK, and A‐BK critically reviewed the manuscript for important intellectual content, writing, and editing.

## FUNDING INFORMATION

This study was funded by University of Oxford.

## CONFLICT OF INTEREST STATEMENT

The authors have no conflicts of interest.

## Data Availability

Data sharing is not applicable to this article as no new data were created or analyzed in this study.
